# Age-related macular degeneration associated polymorphism rs10490924 in *ARMS2* results in deficiency of a complement activator

**DOI:** 10.1186/s12974-016-0776-3

**Published:** 2017-01-05

**Authors:** Sven Micklisch, Yuchen Lin, Saskia Jacob, Marcus Karlstetter, Katharina Dannhausen, Prasad Dasari, Monika von der Heide, Hans-Martin Dahse, Lisa Schmölz, Felix Grassmann, Medhanie Alene, Sascha Fauser, Harald Neumann, Stefan Lorkowski, Diana Pauly, Bernhard H. Weber, Antonia M. Joussen, Thomas Langmann, Peter F. Zipfel, Christine Skerka

**Affiliations:** 1 Department of Infection Biology, Leibniz Institute for Natural Product Research and Infection Biology, Beutenbergstrasse 11, 07745 Jena, Germany; 2 Department of Ophthalmology, Charité Universitätsmedizin Berlin, Augustenburger Platz 1, 13353 Berlin, Germany; 3 Laboratory for Experimental Immunology of the Eye, Department of Ophthalmology, University of Cologne, Joseph-Stelzmann-Str. 9, 50931 Cologne, Germany; 4 Institute of Nutrition, Friedrich Schiller University Jena, Dornburger Str. 25, 07743 Jena, Germany; 5 Institute of Human Genetics, University of Regensburg, Franz-Josef-Strauß-Allee 11, 93053 Regensburg, Germany; 6 Institute of Reconstructive Neurobiology, University of Bonn, Sigmund-Freud-Str. 25, 53127 Bonn, Germany; 7 Department of Ophthalmology, University Hospital Regenburg, Franz-Josef-Strauß-Allee 11, 93053 Regensburg, Germany; 8 Friedrich Schiller University, Fürstengraben, 07743 Jena, Germany

## Abstract

**Background:**

Age-related macular degeneration (AMD) is the leading cause of blindness in developed countries. The polymorphism rs10490924 in the *ARMS2* gene is highly associated with AMD and linked to an indel mutation (del443ins54), the latter inducing mRNA instability. At present, the function of the ARMS2 protein, the exact cellular sources in the retina and the biological consequences of the rs10490924 polymorphism are unclear.

**Methods:**

Recombinant ARMS2 was expressed in *Pichia pastoris*, and protein functions were studied regarding cell surface binding and complement activation in human serum using fluoresence-activated cell sorting (FACS) as well as laser scanning microscopy (LSM). Biolayer interferometry defined protein interactions. Furthermore, endogenous *ARMS2* gene expression was studied in human blood derived monocytes and in human induced pluripotent stem cell-derived microglia (iPSdM) by PCR and LSM. The ARMS2 protein was localized in human genotyped retinal sections and in purified monocytes derived from AMD patients without the ARMS2 risk variant by LSM. ARMS2 expression in monocytes under oxidative stress was determined by Western blot analysis.

**Results:**

Here, we demonstrate for the first time that ARMS2 functions as surface complement regulator. Recombinant ARMS2 binds to human apoptotic and necrotic cells and initiates complement activation by recruiting the complement activator properdin. ARMS2-properdin complexes augment C3b surface opsonization for phagocytosis. We also demonstrate for the first time expression of ARMS2 in human monocytes especially under oxidative stress and in microglia cells of the human retina. The ARMS2 protein is absent in monocytes and also in microglia cells, derived from patients homozygous for the *ARMS2* AMD risk variant (rs10490924).

**Conclusions:**

ARMS2 is likely involved in complement-mediated clearance of cellular debris. As AMD patients present with accumulated proteins and lipids on Bruch’s membrane, ARMS2 protein deficiency due to the genetic risk variant might be involved in drusen formation.

**Electronic supplementary material:**

The online version of this article (doi:10.1186/s12974-016-0776-3) contains supplementary material, which is available to authorized users.

## Background

Age-related macular degeneration (AMD) is a multi-factorial disease and a prevalent cause of visual impairment in developed countries [1]. Genome-wide association studies revealed that variations in or near the complement genes *CFH* [2–5], *CFI* [6], *CFB* [7], and *C3* [8] are significantly associated with AMD. Thus, inappropriate complement activation and innate immunity are linked to the pathogenesis of AMD [1]. The complement system is a major part of innate immunity and plays an essential role in cellular homeostasis, tissue remodeling, as well as in host defense and inflammation [9, 10]. Deregulated complement function or uncontrolled activation due to defective regulation has been implicated in AMD and other diseases including C3-glomerulopathy, paroxysmal nocturnal hemoglobinuria, and systemic lupus erythematosus [9–11].

In addition to mutations in complement genes, a polymorphism (rs10490924) in *ARMS2* shows the highest association with AMD with an estimated relative risk of 8.1 for homozygous carriers [12–14]. The *ARMS2* gene is present only in higher primates [15], and cellular expression and function of ARMS2 are widely unknown. Here, we focused on the role of ARMS2 in AMD pathophysiology and aimed at defining the functional consequences of the AMD associated polymorphism (rs10490924) in *ARMS2*. Our findings demonstrate ARMS2 expression in human monocytes and microglia cells and suggest that ARMS2 functions are directly linked to the complement system, thereby mediating the opsonization of apoptotic and necrotic cells.

## Methods

### Patients

The polymorphism in the *ARMS2* gene, rs10490924 was described as highly associated with both forms of AMD leading to geographic atrophy (dry form) or neo-vascularization (wet form) [12, 13]. Patients diagnosed with the wet form of AMD according to the modified version of AMD study grading system (AREDS) as described previously by Spencer et al. [16] were genotyped for the polymorphisms in the *ARMS2* gene rs2736911, rs10490924, and del443ins54 as described [2, 12]. Genomic DNA was extracted from 10 ml whole blood cells of each patient using the PAX gene blood DNA kit (QiaGen). *ARMS2* was amplified with primers (forward 5′TGTCACCACATTATGTCCC3′ or 5′TGTCACTGCATTCCCTCCTGTCAT3′ and reverse 5′GGCACCACTCCAGAATTT3′ or 5′AAGCTTCTTACCCTGACTTCCAGC3′), and the PCR products were separated by agarose gel electrophoresis, visualized under UV light and subsequently validated by bi-directional sequencing on an automated DNA sequencer (ABI/1130x, Applied Biosystems). According to the presence of the polymorphisms rs2736911, rs10490924, and del443ins54 in the *ARMS2* gene, three groups of genotypes were created (homozygous without these polymorphisms (type I/I), heterozygous for rs10490924 and del443ins54 (type I/II), homozygous for rs10490924 and del443ins54 (type II/II), and homozygous for rs2736911 (type III/III).

### Human donor eyes

Retinal samples of controls and AMD patients were obtained from the Center of Ophthalmology Eye Bank, University of Cologne. Retina 1: type I/I, craniocerebral injury, unknown hour postmortem, age 22. Retina 2: type I/I, intracranial bleeding, 27 h **p**ostmortem, age 82. Retina 3: type I/I, hypoxia brain damage, 4.5 h postmortem, age 53. Retina 4: type II/II, exenteratio orbitae, 8 h postmortem, age 78.

### Cells

CHO-K1 Chinese ovary hamster cells (ATCC-CCL-61), pgsD-677 heparan sulfate deficient CHO cells (ATCC CRL-2244), pgsA-745 xylosyltransferase 1 deficient CHO cells (ATCC CRL-2242), THP-1 human monocytes (ATCC TIB-202), RAW264.7 Mouse leukemic macrophages (ATCC TIB-71), and native RPE cells (InnoProt) were all cultivated according to the costumer’s advise. Human T cells, monocytes, and human erythrocytes were obtained from human blood samples of healthy volunteers. Human T cells, peripheral blood mononuclear cells (PBMCs), and erythrocytes were isolated with micro beads from Miltenyi Biotech, according to the manufacturer’s protocol. Apoptosis of cells was induced by incubation of the cells with 0.4 μg/ml staurosporine for 24 h and necrosis by 1 h at 65 °C. Apoptosis was confirmed by PI and annexin V-pacific blue positive staining (Life Technologies) using flow cytometry. Human microglia cell lines (iPSdM) were generated from induced pluripotent stem (iPS) cell lines obtained by reprogramming skin fibroblasts as previously described [17]. The cells proliferate without addition of growth factors, and they were passaged 1:3 twice a week. The microglia phenotype was confirmed by flow cytometry by expression of CD11b, CD11c, CD14, CD16, CD32, CD36, CD45, CD206, CX3CR1, and TREM2.

### *ARMS2* gene expression (PCR)

Cells (about 1 × 10^6^ from each cell type) with or without stimulation by 400 ng PMA LPS for 24 h or of 10 ml whole blood were harvested and homogenized; total RNA was isolated using the PAX gene blood RNA kit (QiaGen). A 20 ng of isolated RNA was transcribed into cDNA using QuantiTect® Reverse Transcription Kit (QiaGen). cDNA was amplified using Phusion PCR Kit (New England Biolabs), and primers situated in exon 1 (5′TCGGTGGTTCCTGTGTCCTTCATT3′) and exon 2 (5′TCACCTTGCTGCAGTGTGGATGAT3′) of *ARMS2* or for amplification of *actin* (forward 5′ACCAACTGGGACGACAT3′; reverse 5′CTAGAAGCATTTGCGGTG3′). Synthesis was performed by denaturing for 60 s at 96 °C followed by annealing for 30 s at 69 °C (*ARMS2*) or 60 °C (*actin*) and synthesis for 60 s (*ARMS2*) or 120 s (*actin*) at 72 °C for 35 cycles. Extension was performed for 480 s at 72 °C. Amplified PCR products were separated in agarose and visualized under UV light. Bands were excised, purified, and sequenced on an automated DNA sequencer (ABI/1130x, Applied Biosystems). Sequences were aligned to *ARMS2* genomic sequences (NM_001099667).

### Expression and purification of recombinant ARMS2

ARMS2 was recombinant expressed using the *Pichia pastoris* expression system. The expression vector *pPICZB* contained codon usage optimized full length cDNA of the *ARMS2* gene coupled to a *myc* and 6 × *histidine* coding tag for purification (Life Technologies) (Additional file 1: Figure S1). *P. pastoris* cells (strain X33) were transformed with the recombinant expression vector, and ARMS2 expressing clones were selected via zeocin containing selection medium according to the standard protocols. Expressed His-tagged proteins were purified by Ni^2+^-chelate affinity chromatography from the cells [18, 19]. Cell lyses were performed in binding buffer (10 mM Na_2_HPO_4_, 10 mM NaH_2_PO_4_, 10 mM imidazol, 1 M NaCl, 2% (*v*/*v*) Nonidet P-40, 20% (*v*/*v*) glycerol; pH 7.0) together with glass beads (Roth) by FastPrep®-24 (MP Biomedicals) at 4 m/s for 60 s. Protease activity was inhibited by addition of complete EDTA-free (Roche). Protein was eluted from HisTrap™ HP column (GE Healthcare) using elution buffer (10 mM Na_2_HPO_4_, 10 mM NaH_2_PO_4_, 500 mM imidazol, 500 mM NaCl; pH 7.0). Purified ARMS2 was concentrated in storage buffer (10 mM Na_2_HPO_4_, 10 mM NaH_2_PO_4_, 500 mM NaCl; pH 7.0). In addition to the recombinant protein, three ARMS2 peptides were generated. Peptide 1 included amino acids 1 to 40, peptide 2 amino acids 41 to 70, and peptide 3 amino acids 71 to 107 (Jerini Peptide Biotechnologies). Polyclonal ARMS2 antiserum was generated by immunization of rabbits with the here described recombinant ARMS2 protein expressed in *P. pastoris* (Davids Biotechnologies). Generated ARMS2 antiserum (ARMS2_Jena_) was purified with HiTrap Protein A HP 1 ml column (GE Healthcare) and the major epitope of these antibodies determined by pepspot analysis. Peptides of ARMS2 (33 peptides of 13 amino acids with three amino acids overlap) were spotted to a membrane (Jerini Peptide Technologies), incubated with the antiserum and secondary anti-rabbit antibodies for detection. Polyclonal rabbit antiserum generated to the N-terminus of ARMS2 (ARMS2_com_) was purchased from CovaLab. In addition, a mouse monoclonal antibody was generated to the C-terminal peptide 3 of ARMS2 (aa 71–107 = IHTELCLPAFFSPAGTQRRFQQPQHHLTLSIIHTAAR) (ARMS2_mAb_) by standard methods.

Recombinant ARMS2 was separated by SDS-PAGE under reducing conditions (50 mM TRIS-HCL, 1.6% (*w*/*v*) SDS, 7% (*v*/*v*) glycerol, 8 M UREA, 4% (*v*/*v*) β-mercaptoethanol, 0.016% (*w*/*v*) bromophenol blue; pH 6.8) and visualized either by silver staining or immune-blotting using ARMS2_com_, ARMS2_Jena_ antiserum, or mouse α-Penta-His monoclonal antibodies (QiaGen). Mass of whole deglycosylated recombinant ARMS2 was determined by mass spectrometry. To remove the carbohydrate side chains from recombinant ARMS2 (10 μg), the protein was incubated with 0.5 units of PNGase (Roche) for 3 h at 37 °C or the purification tag was cleaved off by enterokinase (New England Biolabs) overnight at RT and repurified with a HisTrap™ HP column.

For immunoprecipitation, monoclonal α-ARMS2 antibody (20 μg) was loaded onto Protein G Magnetic beads (New England BioLabs) for 1 h at 4 °C in binding buffer (20 mM sodium phosphate buffer, pH 7.0). THP-1 cells (1 × 10^7^) were lysed in 1 ml lysis buffer (150 mM NaCl, 1% NP-40, 25 nM Tris-HCl, 1 mM EDTA, 5% glycerol) containing 1 mM PMSF, centrifuged at 16,000 g for 10 min at 4 °C, added to the antibody-loaded beads and incubated on a rotating shaker overnight at 4 °C. After removal of the supernatant, the beads were washed and proteins were eluted from the beads in 30 μl of elution buffer (0.1 M glycine, pH 2.7). The eluted proteins were evaluated for the presence of ARMS2 by SDS-PAGE and mass spectrometry. To show endogenous ARMS2 expression, monocytes were isolated from fresh blood and about 1 × 10^6^ cells were stressed by addition of H_2_O_2_ (0.001–1 mM) for 1 h. Supernatants were replaced by cell culture medium, and cells were incubated for 20 h. Cells were lysed in buffer (50 mM TRIS-HCL, 1.6% (*w*/*v*) SDS, 7% (*v*/*v*) glycerol, 8 M UREA, 4% (*v*/*v*) β-mercaptoethanol, 0.016% (*w*/*v*) bromophenol blue; pH 6.8), centrifuged and supernatants were separated by SDS PAGE, blotted to a membrane and ARMS2 detected with αARMS2_Jena_.

### Mass spectrometry

Recombinant ARMS2 or immunoprecipitated ARMS2 from THP-1 cells were separated by SDS-PAGE and stained with coomassie blue. Protein bands were excised from the gel and washed repeatedly in water and 50 mM NH_4_HCO_3_/acetonitrile 1 + 1 (*v*/*v*) for 15 min. Gel pieces in acetonitrile were dried by vacuum centrifugation. Gel pieces were soaked in 50 mM NH_4_HCO_3_ with 10 mM DTT for 45 min at 56 °C followed by alkylation (50 mM iodoacetamide in 50 mM NH_4_HCO_3_ for 30 min in the dark). Upon washing in 50 mM NH_4_HCO_3_/acetonitrile, gel pieces were incubated in trypsin digestion buffer (20 ng/μl trypsin in 25 mM NH_4_HCO_3_) overnight at 37 °C. The peptides were extracted from the gel plugs by incubation in acetonitrile/trifluoroacetic acid 1 + 1 (*v*/*v*) for 30 min at RT. Extracted peptides (1 μl) were premixed with the same volume of the matrix α-CHCA solution and spotted on a matrix-assisted laser desorption ionization (MALDI) plate. Mass was analyzed using MALDI TOF-mass spectrometer (UltrafleXtreme, Bruker Daltonics, Germany) in the reflector mode with appropriate *m*/*z* range and laser intensity. For analysis of the whole ARMS2 protein, the recombinant protein was deglycosylated and untagged as described above. Analysis of the protein, the instrument was utilized in linear mode. Data analysis was performed with current NCBInr database using Mascot search with a peptide mass tolerance of 100 ppm. The analysis was repeated three times.

### Detection of endogenous ARMS2 by LSM microscopy

For detection of endogenous ARMS2 protein, monocytes of seven ARMS2 genotyped individuals (two (I/I), one (I/II), two (II/II) one (III/III)) were isolated from PBMCs derived from 20 to 40 ml human blood samples. Blood samples were diluted 1:1 in PBS and overlaid with 15 ml Ficoll. After centrifugation at 1600 rpm for 20 min, PBMCs were collected and washed in 10 ml PBS. Monocytes were isolated from PBMCs using a Percoll gradient (3 ml of PBMCs were overlaid by 6 ml of 54% Percoll) centrifuged as above, harvested, and washed in ice-cold PBS. Cells (1 × 10^6^) were transferred into a 4-well slide chamber (Lab-Tek®) and stained for laser scanning microscopy as previously described [19]. Briefly, isolated monocytes or THP-1 cells in chamber slides were fixed with paraformaldehyde (3% at RT for 15 min) permeabilized in Triton X-100 (0.3%) (Roth) 5 min on ice, blocked with FcR blocking reagent (Miltenyi) 10 min at 4 °C and stained with polyclonal rabbit ARMS2 antiserum (αARMS2_Jena_) (1:200), monoclonal ARMS2 antibodies (αARMS2_mAb_) (1:200) followed by incubation with Alexa-647 labeled anti-rabbit or anti-mouse (1:400) for 1 h at RT. DNA was stained with DAPI (10 μg/ml) for 15 min at RT. After washing, the samples were examined by LSM (LSM 510, Carl Zeiss, Jena).

CHO-K1 cells (~1 × 10^5^) were incubated with 10 μg ARMS2 and properdin, either alone or together. After 1 h, cells were washed and placed on a chamber slide (Nunc). After 1 h, adherent cells were fixed with 3% paraformaldehyde for 15 min. CHO surface-bound ARMS2 was visualized using rabbit ARMS2 antiserum (1:100) and Alexa-488 conjugated secondary antibody (1:200; Life Technologies) in assay buffer (1% BSA in RPMI media). Properdin was detected by mouse monoclonal properdin antibody (1:100; QuiDel) and secondary Alexa-647 conjugated antibody (1:200; Life Technologies) in assay buffer. DAPI (Sigma) was used to stain DNA. Images were taken by LSM 710 Meta (Zeiss) and co-localization was analyzed with Zen 2009 software (Zeiss). All incubation steps were done in RPMI media at RT.

### Histology

Eyes were soaked in phosphate buffered saline (PBS) containing 4% formaldehyde followed by punching of a tissue strip ranging from the optic nerve to the fovea/parafovea which was then processed for paraffin embedding. Sections of 12 μm thickness derived from 2 genotyped individuals (genotype (I/I) and (II/II)) were obtained and subjected to immunohistochemical staining. After de-paraffinization by a series of graded ethanol slides were placed in a 97 °C hot water bath in antigen retrieval buffer (0.05 M Tris buffered saline, 0.05% Tween-20, pH 9.0) for 20 min. Slides were then washed with 1% goat serum in 1xPBS-T (PBS with 0.4% Triton-X) for 10 min for two times. To prevent unspecific binding, sections were covered with blocking buffer (5% goat serum in 1xPBS-T) in a humidified chamber and incubated for 30 min at room temperature. Primary antibodies included anti-ARMS2 (1:200, rabbit polyclonal), anti-CD68 (1:200, monoclonal rat anti-mouse, AbDSerotec), and anti-CD68 (1:200, monoclonal mouse anti-human, Dako). Primary antibodies were incubated overnight at 4 °C. After the two washing steps with 1% serum PBS-T, sections were incubated with labeled secondary antibodies coupled to Alexa-488 (green) or Alexa-594 (red) (Jackson ImmunoResearch, West Grove, PA, USA). Retinal nuclei were counter-stained with DAPI and mounted in DAKO fluorescent mounting medium (Dako Deutschland GmbH, Hamburg, Germany) and analyzed on an Axioskop 2 MOT plus Apotome microscope (Zeiss, Jena, Germany).

### Binding analyses

#### ELISA

##### ARMS2 binding to complement proteins

C3, C3b, C3c, C3d, iC3b, factor B, properdin (all Complement Technologies) and human serum albumin (HSA) (Nutritional Biochemicals) (each 500 ng in PBS) were coated onto MediSorp microtiter plates (Nunc) over night at 4 °C. Wells were blocked with buffer I (AppliChem) for 2 h at RT and incubated with 500 ng ARMS2 in assay buffer (2% (*w*/*v*) BSA, 0.05% (*v*/*v*) Tween-20 in PBS) for 1 h at RT. ARMS2 was detected with ARMS2 antiserum diluted 1:1000 and a secondary HRP conjugated antibody 1:2000 in CrossDown buffer (AppliChem) at 492 nm.

##### Properdin binding to ARMS2

Recombinant ARMS2 or HSA (each 500 ng in PBS) was coated onto MaxiSorp microtiter plates (Nunc) over night at 4 °C. Wells were then washed with PBS + 0.05% (*v*/*v*) Tween-20 and blocking was performed with blocking buffer (5% (*w*/*v*) BSA, 0.05% (*v*/*v*) Tween-20 in PBS) for 2 h at RT. After another washing step, wells were incubated with increasing amounts of properdin (12.5–100 nM) in assay buffer (2% (*w*/*v*) BSA, 0.05% (*v*/v) Tween-20 in PBS). After incubation for 1 h at RT, wells were washed and bound properdin was detected with a goat properdin antiserum diluted 1:2000 and a secondary horse radish peroxidate (HRP)–conjugated antibody diluted 1:2000 in assay buffer. Multiscan Ascent ELISA Reader measured absorption at 492 nm. To determine the interaction domain in ARMS2 responsible for properdin binding to ARMS2, peptide 1, peptide 2, and peptide 3 (each 500 nM) were coated to the microtiter plate, incubated with 2 μM properdin (TECOmedical GmbH) in PBS and binding of properdin was detected as described before. Primary monoclonal properdin antibody (1:1000) [20] and secondary anti mouse 1:2000 (Dako, Denmark) were used for detection.

The binding affinity of properdin to ARMS2 was measured using biolayer interferometry on a BLITZ system (Forte Bio). His-tagged ARMS2 was loaded onto Ni^2+^ NTA biosensors (Forte Bio) in assay buffer (PBS (Lonza) with 0.1% (*m*/*v*) gelatin). Loaded biosensors were blocked using assay buffer with 10 μg/ml biocytin (Sigma-Aldrich) for 60 s. After washing, the biosensors were dipped in 4-μl properdin solution with concentrations varying from 70 to 1800 nM properdin. The *k*_D_ was determined by fitting data to a 1:1 model algorithm with the BLITZ software.

##### Recruitment of C3b by ARMS2 bound properdin

Properdin (100 nM) was bound to immobilized recombinant ARMS2 as described before. Wells were washed with assay buffer (2% (*w*/*v*) BSA, 0.05% (*v*/*v*) Tween-20 in PBS) and incubated with increasing amounts of C3b (4–12 nM) in assay buffer for 1 h at RT. Unbound C3b was removed by additional wash steps, and binding was detected at 492 nm by mouse C3d monoclonal antibody (1:2000, Quidel) and secondary HRP-conjugated antibody (1:2000) in assay buffer.

##### Ba activities

ARMS2 (500 nM) was incubated with 20% NHS in Mg^2+^EGTA buffer (1% (*w*/*v*) BSA, 20 mM HEPES, 144 mM NaCl, 7 mM MgCl_2_, 10 mM EGTA, pH 7.4) for 15 min at 37 °C. The generated Ba product was analyzed at 540 nm using a Ba MicroVUE ELISA Kit (QuiDel).

#### Flow cytometry

All fluoresence-activated cell sorting (FACS) analyses were performed with a LSR II flow cytometer (BD Science); 10,000 cells were evaluated and data were calculated using FlowJo Software (Tree Star).

##### ARMS2 deposition

CHO-K1, pgsD-677, pgsA-745 (each 1 × 10^6^), native RPE (1 × 10^5^), human erythrocytes (each 1 × 10^7^) as well as isolated naive T cells (1 × 10^6^) were incubated with increasing amounts of ARMS2 (10–500 nM) in assay buffer (1% (*w*/*v*) BSA in PBS) for 1 h on ice. ARMS2 binding was detected with rabbit ARMS2 antiserum (1:200) and an Alexa-647 conjugated antibody (Life Technologies) (1:400). Similarly, factor H (10–500 nM, Comptech) was bound to CHO-K1 cells.

##### Heparan sulfate competition assay

Recombinant ARMS2 (500 nM) was incubated with 1 × 10^7^ heparin coated beads (GE Healthcare) and increasing amounts of heparan sulfate (0–12 mM, Sigma) in assay buffer (1% (*w*/*v*) BSA in PBS) for 1 h on ice. ARMS2 binding was detected with rabbit ARMS2 antiserum 1:200 and Alexa-647 conjugated antibodies (Life Technologies) 1:400. Factor H (500 nM, Comptech) was incubated with the beads and detected with factor H antiserum (Comptech).

##### C3b deposition

Complement activation assays were performed as previously described [19]. CHO-K1 and pgsA-745 (each 1 × 10^6^) were incubated in 20% NHS, or heat-inactivated 20% NHS (hiNHS) (30 min at 56 °C) forms healthy human donors in Mg^2+^EGTA buffer (1% (*w*/*v*) BSA, 20 mM HEPES, 144 mM NaCl, 7 mM MgCl_2_, 10 mM EGTA, pH 7.4) for 45 min at 37 °C. C3b deposition was determined by flow cytometry using mouse α-C3b monoclonal antibody (FitzGerald) diluted 1:100 and secondary Alexa-647 conjugated antibodies (Life Technologies) diluted 1:200 in Mg^2+^EGTA buffer for 1 h on ice. Deposition was normalized and NHS alone defined as 100%. Influence of ARMS2, factor H (CompTech), or HSA (Nutritional Biochemicals) on C3b deposition was evaluated, after preincubation of either cells (1 h on ice) or serum (15 min at 37 °C) with increasing amounts of each protein (100 to 750 nM).

##### Properdin deposition

CHO-K1 cells (1 × 10^6^) were preincubated with 500 nM ARMS2 for 1 h on ice. Washed cells were incubated either in 20% NHS diluted with Mg^2+^EGTA buffer (1% (*w*/*v*) BSA, 20 mM HEPES, 144 mM NaCl, 7 mM MgCl_2_, 10 mM EGTA, pH 7.4) or EDTA buffer (1% (*w*/*v*) BSA, 20 mM HEPES, 144 mM NaCl, 10 mM EDTA, pH 7.4) for 45 min at 37 °C or with 100 nM purified properdin (CompTech) in PBS containing 1% BSA for 1 h at RT. Properdin deposition was analyzed by flow cytometry using a properdin monoclonal antibody (QuiDel) (1:200) followed by an Alexa-647 conjugated antibody (Life Technologies) (1:400).

### Statistical analysis

Significant differences between two groups were analyzed using the Student’s two-tailed *t* test. Values of *p < 0.05, **p < 0.01, ***p < 0.001 were considered statistically significant.

## Results

### Human monocytes and iPS-derived microglia cells express ARMS2

**Fig. 1:**
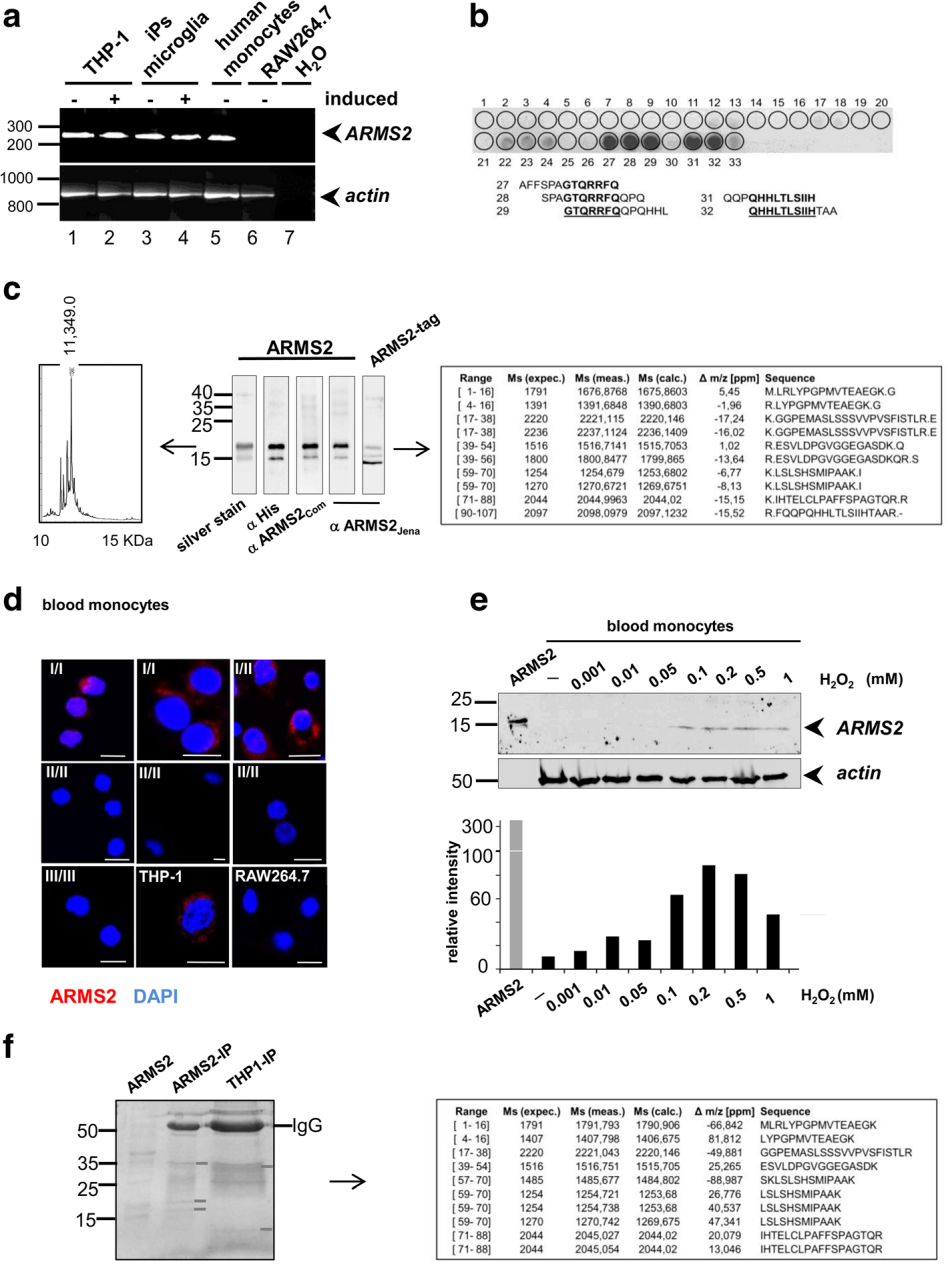
ARMS2 expression in human monocytes and microglia cells. **a** Transcription of *ARMS2* in uninduced or PMA (400 ng, 24 h) induced THP-1 monocytes, mouse RAW 264.7 cells, microglial cells stimulated with LPS (5 ng/ml, 24 h) or blood-derived human monocytes. **b** New polyclonal antiserum generated to recombinant ARMS2 detects the C-terminal part of ARMS2 as indicated by the *dark spots*. Peptides of ARMS2 (33 amino acids with an overlap of 3 amino acids) were spotted to a membrane and incubated with the ARMS2 antiserum. **c** Recombinant ARMS2 expressed in *P. pastoris* and purified by Ni^2+^ chromatography appears as monomeric protein of about ~15/17 kDa identified by silver staining or Western blot analysis using either monoclonal penta-Histidin, purchased polyclonal ARMS2 antiserum (αARMS2_Com_) or polyclonal ARMS2 antiserum (αARMS2_Jena_), generated to the recombinant protein) as indicated. The ARMS2 protein without purification tag showed a mobility of about 11 kDa. Mass spectrometry of the deglycosylated 17 kDa ARMS2 band revealed ARMS2 peptides (raw data). MS of the whole protein showed a protein with a mass of 11.349 kDa. **d** ARMS2 is present in monocytes with one or two copies of the non-risk ARMS2 variant, but is absent in cells with the homozygous rs10490924 (II/II) or rs2736911 (III/III) polymorphism. ARMS2 is detected in the cytoplasm of THP-1 cells (I/II), but ARMS2 is absent in RAW264.7. cells. Cells in (**d**) were permeabilized, stained for ARMS2 (*red*) or DNA (*blue*) and visualized by laser scanning microscopy. *Scale bar* = 10 μm. **e** Blood-derived monocytes express ARMS2 upon oxidative stress. Cells were incubated for 1 h in medium with H_2_O_2_ (0.001–1 mM), for another 20 h in normal medium, lysed in loading buffer, and proteins were separated by SDSPAGE and immunoblotted using αARMS2_Jena_. Densitometric analysis is shown in the graph below. The same blot was also stained for β-actin as a loading control. **f** Immunoprecipitation of ARMS2 from THP-1 cell lysate (1 × 10^7^) with new monoclonal ARMS2 antibodies. Eluted proteins were separated by SDS-PAGE, stained with coomassie blue and single bands (*white bars*) investigated by mass spectrometry. ARMS2 peptides from five immunoprecipitations are shown. Precipitation of recombinant ARMS2 revealed ARMS2 petptides (data not shown). Experiments shown in **a** to **e** are representatives of three independent experiments. ∆*m*/*z* [*ppm*] mass tolerance, *ARMS2-IP* imunoprecipitation of recombinant ARMS2, *THP1-IP* ARMS2 immunoprecipitated from THP-1 cells

A typical hallmark of AMD is the accumulation of cellular material in the form of drusen in the macular region of the retina [1]. Normally, microglia cells, the resident macrophages of the retina, take up modified or dead cells by phagocytosis and the uptake of C3b opsonized cellular debris is markedly enhanced [21, 22]. Since infiltration of inflammatory monocytes is reported in AMD retinas [23], we first asked whether monocytes express ARMS2 and determined ARMS2 mRNA expression in these cells. A specific RT-PCR amplicon was generated with cDNA derived from the human monocytic THP-1 cells and also from human primary monocytes (Fig. 1a). DNA sequencing confirmed that this amplicon represented *ARMS2* (data not shown), thus demonstrating expression of *ARMS2* in human monocytes. Next, we analyzed whether microglia cells, the resident immune cells of the brain and the retina, [24] express ARMS2 transcripts. To circumvent the viability problems with human postmortem microglia, *ARMS2* transcription was analyzed in microglia cells derived from human induced pluripotent stem cells (iPSdM) [17]. Using the same *ARMS2* specific primers as before, a specific *ARMS2* amplicon was generated with microglia-derived cDNA (Fig. 1a). *ARMS2* expression was previously shown in the retina, placenta, and whole blood [2, 12, 25]. Here, we additionally identified *ARMS2* gene expression in human blood monocytes and microglia cells.

### Expression of recombinant ARMS2 and generation of antibodies

To detect the endogenous ARMS2 protein in monocytes, a new polyclonal rabbit ARMS2 antiserum was raised against a recombinant histidine-tagged ARMS2 protein expressed in *P. pastoris*. The polyclonal antiserum was characterized by the identification of the main epitope in ARMS2 bound by the antibodies. Therefore, 33 ARMS2 peptides each 13 amino acid long and with an overlap of three amino acids were spotted to a membrane and subsequently stained with the polyclonal ARMS2 antiserum. The antibodies bound predominantly to the C-terminal end of ARMS2 (Fig. 1b). When separated by SDS-PAGE under reducing conditions, recombinant ARMS2 appeared with mobilities of 17 and 15 kDa by immune staining using the new antiserum and also with a commercial ARMS2 antiserum or a His-tag mAb (Fig. 1c). The 17 kDa protein band was cut out, deglycosylated, digested by trypsin, and then analyzed by mass spectrometry. All peptides derived from the 17 kDa band covered regions (95%) of ARMS2. MS of the untagged, deglycosylated protein revealed a dominant protein peak with a mass of 11.349 Da.

### Monocytes derived from AMD patients with the ARMS2 risk haplotype lack the ARMS2 protein

The ARMS2 risk variant is defined by the polymorphism rs10490924, which is linked to an indel mutation (del443ins54) in the 3′ untranslated region of the *ARMS2* gene [12]. The *ARMS2* indel mutation is considered to lead to *ARMS2* mRNA instability, as this mutation deletes the poly A signal and integrates two AUUUA motifs that mediate rapid mRNA turnover. To determine whether this *ARMS2* risk variant (rs10490924 with del443ins54) affects *ARMS2* expression, we determined *ARMS2* expression on the protein level in peripheral blood monocytes isolated from genotyped AMD patients. Monocytes of three different genotypes were compared as follows: type I/I harbors two non-risk alleles of *ARMS2*. type I/II has one *ARMS2* risk allele (rs10490924 with del443ins54) and a non-risk allele, and type III has two alleles of the ARMS2 risk variants (rs10490924 with del443ins54). Blood monocytes of the three genotypes were isolated from AMD patients, fixed in chamber slides, permeabilized, and stained with ARMS2 antiserum and with a monoclonal ARMS2 antibody. The ARMS2 protein was identified in the cytoplasm of monocytes harboring one or two alleles of the ARMS2 non-risk variant (i.e., type I/I or I/II) (Fig. 1d). In contrast, monocytes derived from the three patients with two risk alleles (II/II) showed no ARMS2 protein staining (Fig. 1c). Also, monocytes derived from a single patient homozygous with an *ARMS2* stop mutation (rs2736911, genotype III/III) lacked the ARMS2 signal. To verify ARMS2 presence in the monocytes, ARMS2 was also stained in monocytic cells THP-1 using polyclonal antiserum (αARMS2_Jena_). Again, single spots were detected in the cytoplasm of the cells (Fig. 1d). In contrast, macrophages of murine origin lacked an ARMS2 signal. As the signal of ARMS2 in monocytes was very weak, we tested whether ARMS2 expression is enhanced under stress signals. Therefore, human blood monocytes were challenged for 1 h with H_2_O_2_ and grown over night and lysates investigated for ARMS2 expression by Western blot analysis. Interestingly, ARMS2 expression increased in monocytes upon oxidative stress (Fig. 1e). Having shown that ARMS2 is expressed in THP-1 cells, immunoprecipitation was performed combined with mass spectrometry to detect the endogenous ARMS2 protein. A new generated monoclonal ARMS2 antibody was bound to a protein G column, incubated with THP-1 whole cell lysate, washed and antibody bound proteins were eluted from the column. After separation via SDS PAGE and coomassie blue staining, single bands were extracted from the gel (Fig. 1f). Mass spectrometry revealed ARMS2 peptides in the cell lysate of THP-1 cells (Fig. 1f) and in probes derived from precipitated recombinant ARMS2 (not shown). Altogether, the results demonstrate for the first time ARMS2 protein expression in monocytes and that the risk variants of ARMS2 lead to ARMS2 deficiency in these cells.

**Fig. 2:**
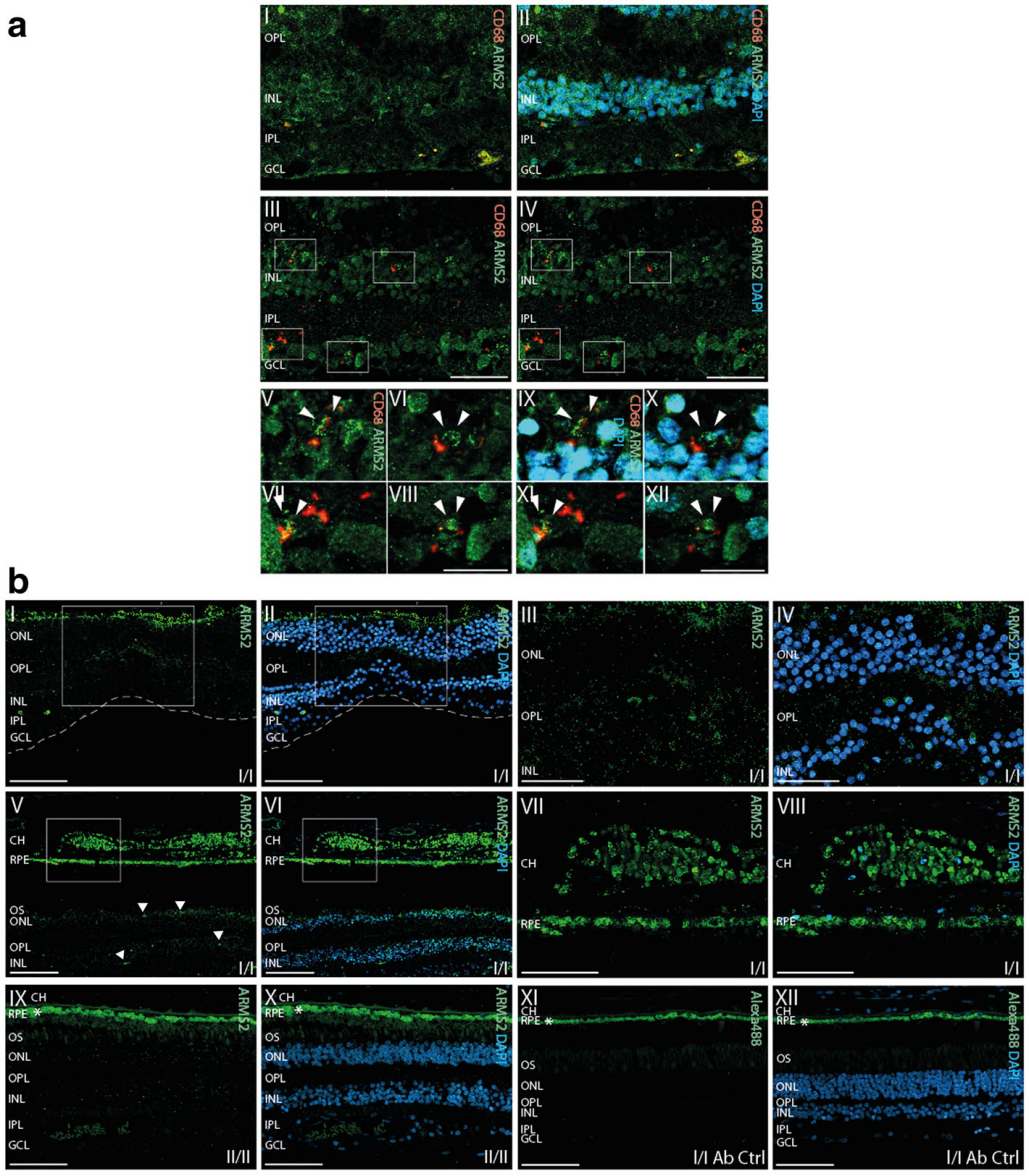
ARMS2 expression by retinal microglia cells. **A I**, **II** Co-labeling of ARMS2 (*green*) and the macrophage/microglia marker CD68 (*red*) in the human retina shows co-localization (*white arrowheads*) of ARMS2 and CD68 indicating ARMS2 expression by retinal microglia cells/macrophages in the human retina. Note that blood monocytes in retinal vessels (*dotted lines*) co-labeled for ARMS2 and CD68. **A II**, **IV** Co-labeling of ARMS2 and CD68 shows that retinal microglia are associated with cells of the inner retina that contain high amounts of ARMS2 on their cell surfaces (*white squares*). **A IV–XII** Magnifications of squared areas in (**II** and **IV**) show close association of microglia with retinal neurons and strong ARMS2 immunoreactivity (*white arrowheads*). *Left panels* (**V**–***VIII***) show ARMS2 and CD68. *Right panels* (**A IX–XII**) show merged pictures of ARMS2 and CD68 and DAPI. *INL* inner nuclear layer, *IPL* inner plexiform layer, *GCL* ganglion cell layer. *Scale bar* (**A I–IV**) 50 μm; *scale bar* (**A V–XII**) 25 μm. **B I**, **II** ARMS2 staining of retinal cross section through the fovea of a human donor with haplotype (I/I). The *white squares* indicate the area of the macular that is magnified in (**B II**, **IV**). **B V**, **VI** ARMS2 immunoreactivity in retinal cross section of parafoveal areas obtained from a human donor with haplotype (I/I). The *white squares* indicate the area of RPE and choroid magnified in (**B VII**,**VIII**). ARMS2 staining in the choroid is presumably confined to monocytes and choroidal macrophages, in the retina to microglia cells (*arrows*) (**B IX**, **X**). Retinal cross section from a human donor with haplotype (II/II) that is devoid of ARMS2 immunoreactivity also in the choroid. **B XI**, **XII** Control stainings lacking primary antibody. *Green autofluorescence lining at the top of the images* is derived from RPE (*asterisk*). *Scale bar* (**B I**, **II**, **V**, **VI**, **IX**, **X**) 50 μm; *scale bar* (**B III**, **IV**, **VII**, **VII**, **XI**, **XII**) 25 μm

We next analyzed ARMS2 expression and localization in retinal tissue and tried to identify the responsible cells in the human retina, which express the protein. To this end, retinal tissue cross sections from genotyped individuals were analyzed by histology using the new ARMS2 antiserum. ARMS2 was identified in stained cross sections of the retina derived from individuals with the I/I genotype (Fig. 2A, B I–IV), but ARMS2 was not detectable in sections of the II/II genotype (one representative of three stainings is shown, Fig. 2B IX, X). ARMS2 immunoreactivity appeared in the inner retinal layer as a speckled pattern and was also seen in the choroid (Fig. 2A, B V–VIII), which is in agreement with the presence of ARMS2 in monocytes. As iPS-derived microglia cells express the *ARMS2* gene (Fig. 1a), we aimed to demonstrate ARMS2 expression also for the resident retinal microglia. The surface marker CD68 identified several microglia cells in the cross-section that were ARMS2 positive (Fig. 2A I–IV). The CD68 positive ARMS2 expressing microglia cells were frequently associated with neurons that were highly decorated with ARMS2 (Fig. 2A V–VII). Thus, retinal microglia cells with the genotype I/I very likely express and secrete ARMS2, which then binds to neurons.

### ARMS2 binds to cell surfaces

**Fig. 3:**
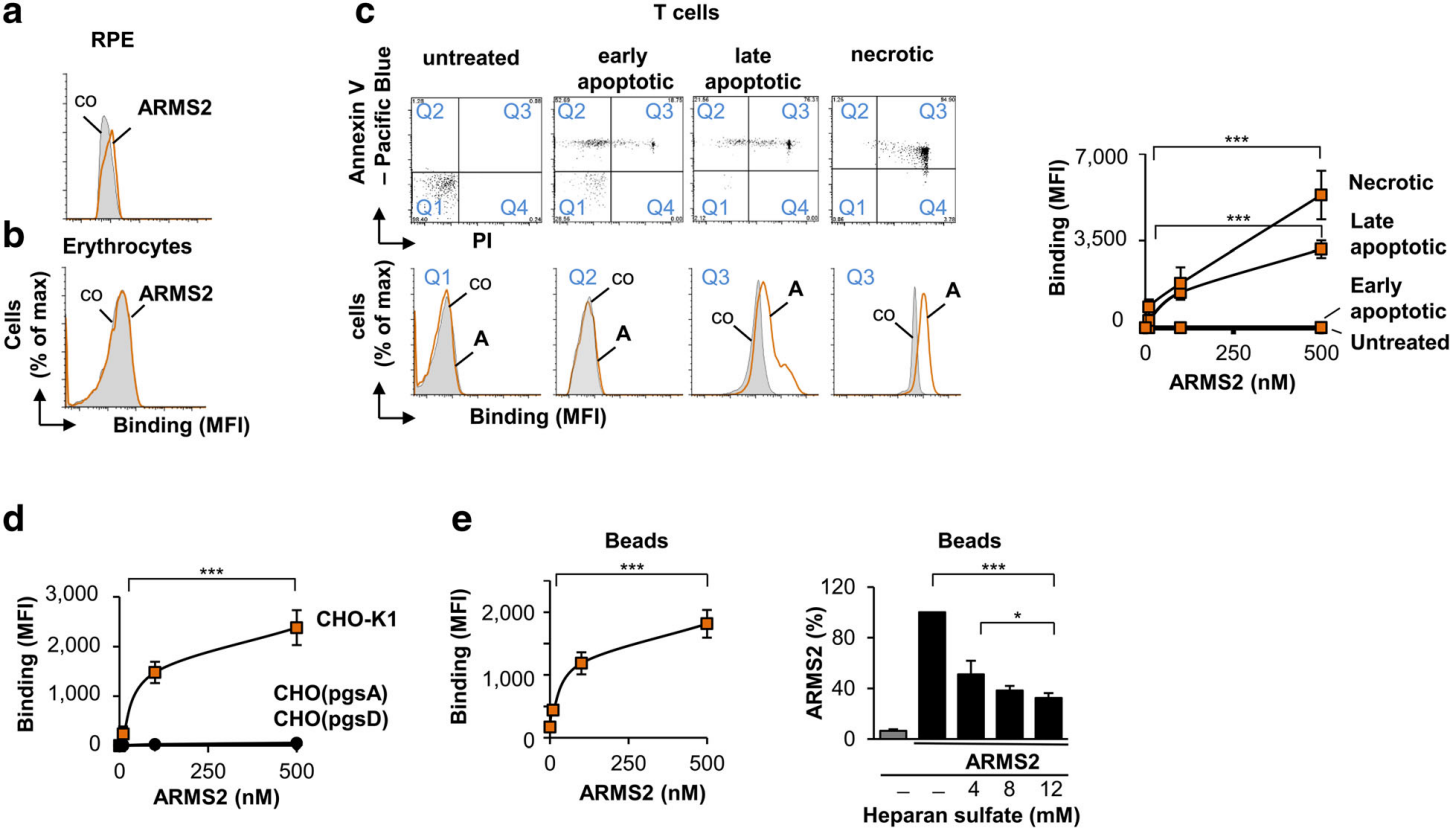
ARMS2 binding to cell surfaces. **a** Binding of ARMS2 (500 nM, 1 h) to naive RPE-cells and (**b**) human erythrocytes. **c** ARMS2 binding to untreated (*n* = 3), early apoptotic (*n* = 4), late apoptotic (*n* = 3), and necrotic (*n* = 4) T cells. T cells were gated according to their staining with annexin V-pacific blue and PI (*upper panels*), flow cytometry of indicated gates are shown (*lower panels*). ARMS2 bound in a dose-dependent manner (***p = 0.0001 for necrotic and ***p = 0.0002 for late apoptotic cells, each 10 nM versus 500 nM ARMS2). Background binding of the antibody to the cells is marked (*co*). **d** ARMS2 binding for 1 h to CHO-K1 (*n* = 4), CHO_pgsA_, or CHO_pgsD_ cells (each *n* = 5). Flow cytometry experiments show the median MFI values ± s.d. using ARMS2 antiserum (***p = 0.0001, 10 nM versus 500 nM ARMS2). **e** ARMS2 binding to heparin coated beads (*n* = 3, median MFI values ± s.d., ***p = 0.0002) and in competition with free heparan sulfate (4–12 mM, *n* = 3, ***p = 0.0002, 0 mM versus 12 mM heparan sulfate, and **p = 0.016, 4 mM versus 12 mM heparan sulfate). Competition is shown in percent of binding of ARMS2 to the beads alone (100%)

To further define the role of the ARMS2 protein, we followed up on the observation of ARMS2 deposited on neurons of the retina and investigated whether ARMS2 binds to cell surfaces using flow cytometry. Purified recombinant ARMS2 did not bind to RPE cells (Fig. 3a) or to human erythrocytes (Fig. 3b). To test if ARMS2 binds specifically to modified human surfaces, we measured ARMS2 binding to naïve, apoptotic, or necrotic human T cells. Apoptotic or necrotic T cells were identified as annexin V and PI positive cells by flow cytometry. In this set up, ARMS2 bound to late apoptotic and necrotic T cells but did not bind to naïve or to early apoptotic T cells (Fig. 3b). This surface attachment is in agreement with the reported ARMS2 binding to extracellular matrix proteins [2, 26] and the localization of ARMS2 close to the drusen, which represent accumulated cellular debris as shown by proteome analysis and immune staining [26, 27].

### ARMS2 binds to heparan sulfate

To identify cellular ARMS2 ligands, we studied whether ARMS2 cell surface binding is mediated by surface exposed glycosaminoglycans (GAGs). To this end, we analyzed ARMS2 binding to CHO-K1 (Chinese Hamster Ovary) cells, which expose sulfated GAGs and also to two mutant lines, which lack surface heparan sulfates. ARMS2 bound to wild type CHO-K1 cells but did not bind to the mutant CHO_pgsA_ or to CHO_pgsD_ cells (Fig. 3d). GAGs represent complex polysaccharides and are exposed at the surface of mammalian cells; GAGs sulfation provides a recognition signal for proteins [28]. To confirm ARMS2 interaction with heparan sulfate in the absence of other cell surface ligands, we studied ARMS2 binding to heparin-coated beads. ARMS2 bound to the coated beads, and binding was blocked when ARMS2 was incubated with heparan sulfate prior to binding (Fig. 3e). Thus, ARMS2 binds to cell surfaces via GAGs.

### Cell surface bound ARMS2 enhances complement activation

**Fig. 4:**
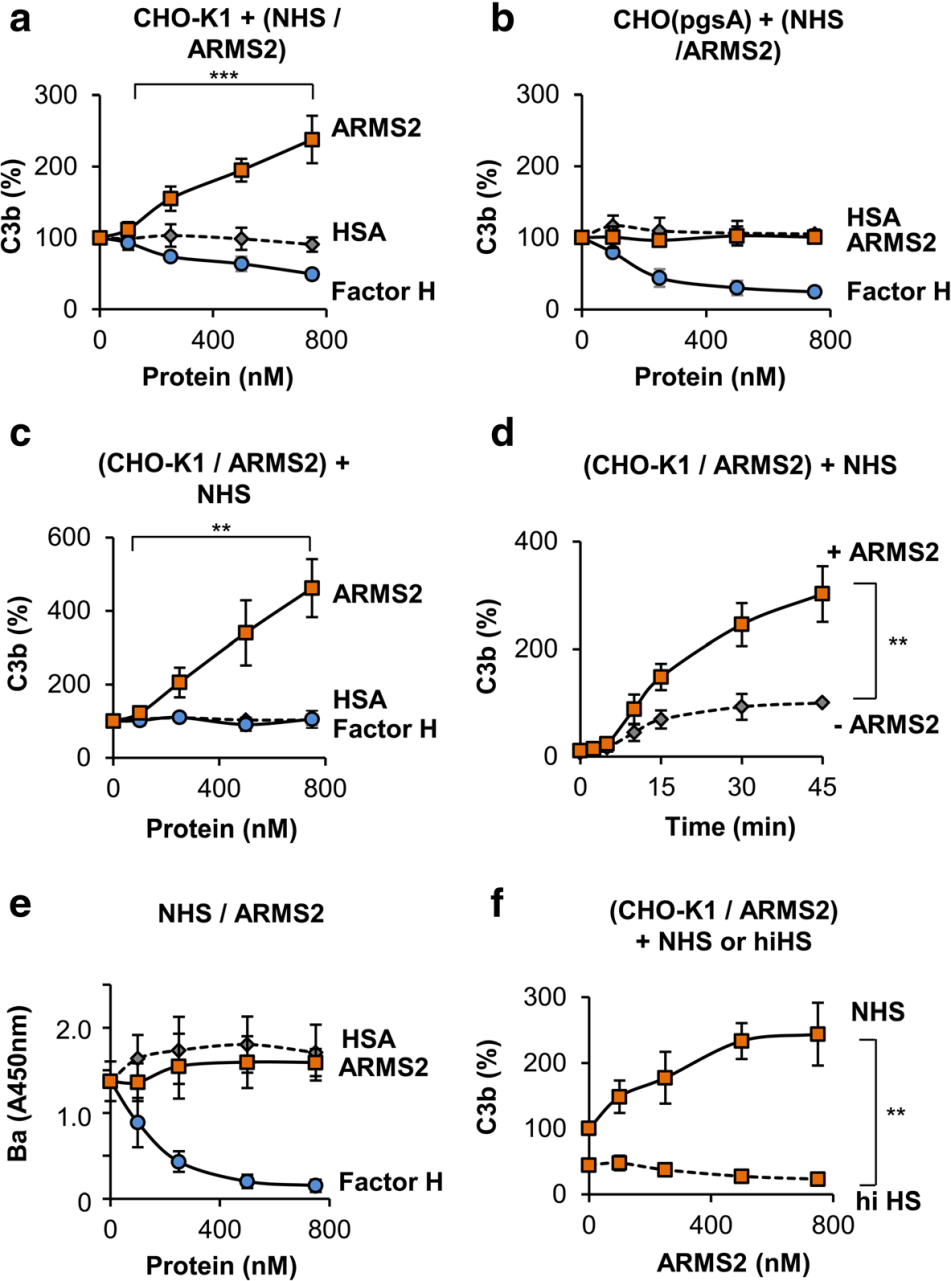
ARMS2 functions as complement activator on the cell surface. **a** C3b deposition on CHO-K1 cells is enhanced after incubation for 45 min in 20% NHS preincubated with ARMS2 (100–750 nM) (*n* = 4, **p = 0.0021, 100 nM versus 750 nM). Human serum albumin (HSA) had no and factor H an inhibiting effect on C3b deposition. C3b deposition on cells in NHS alone was set as 100%. **b** C3b deposition on CHO_pgsA_ cells is not enhanced after incubation in 20% NHS with ARMS2, factor H or HSA (each 100–750 nM) (each *n* = 3). C3b deposition on cells in NHS alone was set as 100%. **c** Deposition of C3b is increased on ARMS2 (100–750 nM) coated CHO-K1 cells (*n* = 4, **p = 0.0021) after incubation in 20% NHS. HSA or factor H does not enhance C3b binding. **d** ARMS2 (500 nM) attached to CHO-K1 cells and incubated for 45 min in 20% NHS enhanced C3b deposition over time as compared to cells alone in 20% NHS (latter was set as 100%). *Error bars* show median MFI values ± s.d. (*n* = 3, **p = 0.0011, NHS versus NHS + ARMS2). **e** ARMS2 (100–750 nM) added to 20% NHS without cells did not enhance Ba generation, as determined by ELISA. Data represent mean values ± s.d. (*n* = 3). **f** Deposition of C3b on ARMS2 (100–750 nM) coated CHO-K1 cells is enhanced in 20% complement active NHS incubated for 45 min but not in heat inactived NHS (30 min, 56 °C). Data represent median MFI values ± s.d. in % (NHS alone was set as 100%) (*n* = 3, **p = 0.0014, NHS versus hiHS at 750 nM ARMS2)

Complement is involved in the pathogenesis of AMD as revealed by genetic association studies and also by the presence of complement products in drusen [1, 9, 10, 27]. Given the key role of deregulated complement in AMD pathophysiology, we postulated that ARMS2 influences complement activation. To test this hypothesis, ARMS2 was added to normal human serum (NHS) and this mixture was then added to CHO-K1 cells. Following incubation and washing, surface C3b deposition was determined by flow cytometry. Addition of NHS to CHO-K1 cells induced complement activation, which was set as 100%. However, ARMS2, when added to NHS, enhanced C3b opsonization of CHO-K1 cells and this effect was dose-dependent (Fig. 4a). In contrast, no enhanced C3b depositon was observed for the mutant line CHO_pgsA_ cells that do not bind ARMS2 (Fig. 4b). C3b opsonization was even more pronounced when ARMS2 was attached to the surface of CHO-K1 cells prior to addition of NHS (Fig. 4c), and complement activation was followed over a time period of 45 min. ARMS2 coated to CHO-K1 cells increased C3b deposition by threefold (Fig. 4d) as compared to cells coated with HSA. This increase of C3b surface decoration suggested that ARMS2 attached to a cell surface activates the amplification loop of complement. In contrast to ARMS2, factor H blocked complement activation in this setup. Remarkably, ARMS2, when added to NHS as a fluid phase protein, did not affect complement activation and in this case, no complement cleavage products like Ba were generated (Fig. 4e). As expected, factor H, when added to NHS in fluid phase, reduced Ba levels. ARMS2 on the surface of CHO-K1 and then incubated with heat-inactivated serum (hiHS) did not affect C3b deposition (Fig. 4f). Thus, ARMS2 is a complement activator that acts exclusively on surfaces.

### ARMS2 binds the human complement activator properdin

**Fig. 5:**
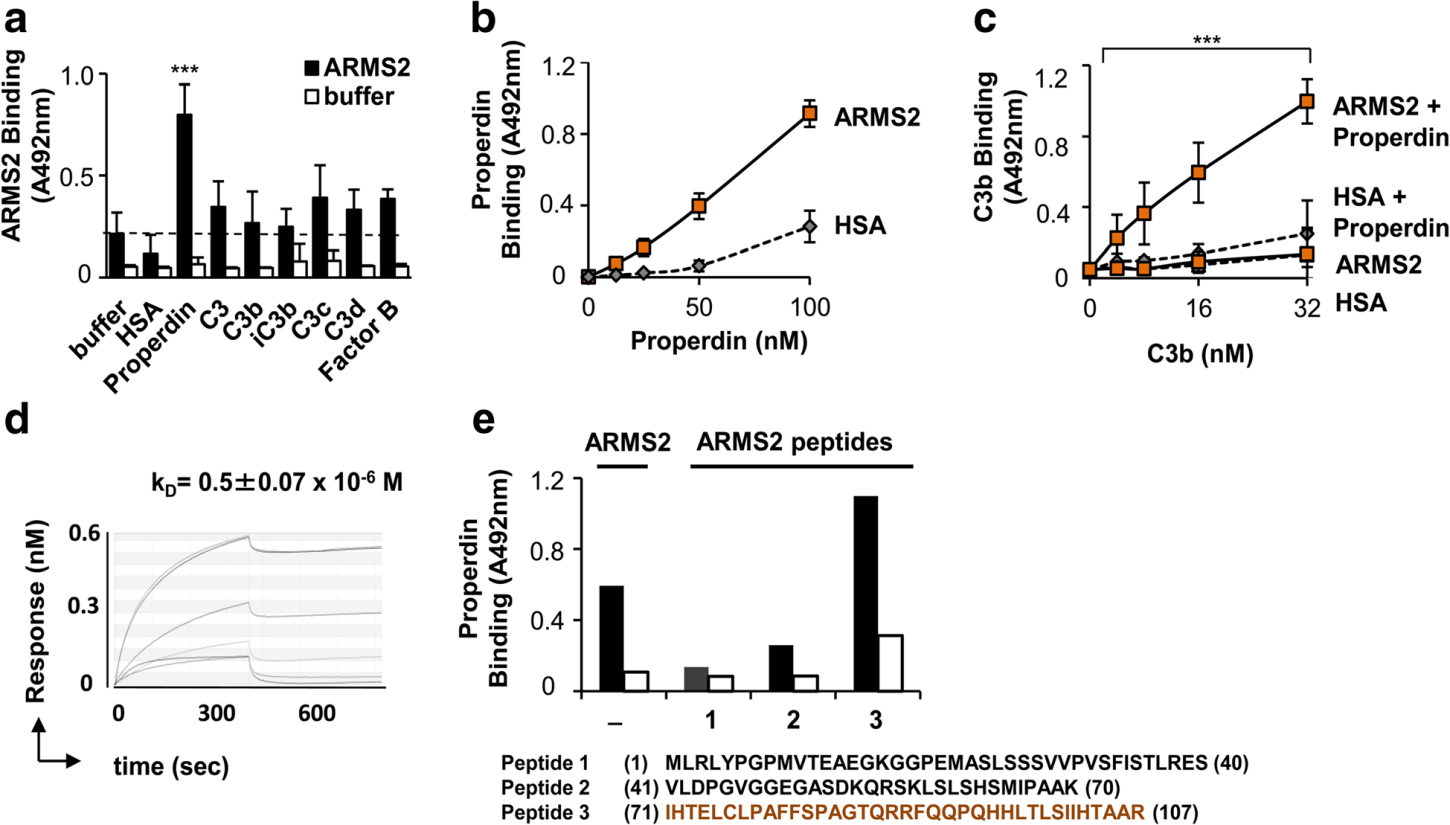
ARMS2 interacts with properdin. **a** Binding of ARMS2 (500 ng, 1 h) to immobilized C3 convertase components properdin, C3, C3b, iC3b, C3c, C3d, and factor B (each 500 ng). *Error bars* represent mean ± s.d. (*n* = 4, ***p = 0.0001, properdin versus buffer). A *stippled lane* indicates background binding. **b** Properdin (12.5–100 nM) binding to immobilized ARMS2 or HSA. *Error bars* represent mean ± s.d. (*n* = 3, **p = 0.001, 12.5 nM versus 100 nM properdin). **c** C3b (4–32 nM, 1 h) binding to properdin (100 nM) attached to immobilized ARMS2 is increased but not to immobilized ARMS2 alone. (*n* = 3, ***p = 0.001, 4 nM versus 32 nM C3b). **d** Affinity constant of the interaction between ARMS2 and properdin was determined by biolayer interferometry. Recombinant ARMS2 was immobilized to the biosensor, and interaction of properdin was determined (mean value ± s.d. of two independent experiments). **e** Recombinant ARMS2 binds properdin within the C-teminal domain. Properdin binding was evaluated to three peptides of ARMS2 immobilized to the plate. Amino acid sequences of the peptides are indicated

To determine how ARMS2 activates complement, binding of ARMS2 to the components of the C3 convertase and to C3 activation products was evaluated. ARMS2 did not bind to C3, to factor B, or to the cleavage products C3b, iC3b, and C3d (Fig. 5a). However, ARMS2 bound to properdin, the only known complement activator [29, 30, 31]. ARMS2 binding to properdin was dose-dependent (Fig. 5b).

To clarify whether ARMS2-properdin complexes recruit C3b, binding of C3b to preformed ARMS2-properdin complexes was followed. ARMS2 was immobilized to a microtiter plate, properdin was attached, and subsequently, C3b was added. Purified C3b bound to the immobilized ARMS2-properdin complex as determined by ELISA, and binding was dose-dependent (Fig. 5c). Thus, surface-bound ARMS2 binds properdin and subsequently, ARMS2-properdin complexes recruit C3b. These results are in agreement with enhanced C3b opsonization when ARMS2 was coated onto CHO-K1 cells prior to challenge with complement active NHS (Fig. 4a). The ARMS2-properdin interaction was confirmed by biolayer interferometry. Recombinant ARMS2 was immobilized to a Ni^2+^ NTA biosensor, and purified properdin was added as analyte. In this set up, properdin bound to immobilized ARMS2 with a *k*_D_ = 0.5 ± 0.07 × 10^−6^ M (Fig. 5d). In order to localize the domain in ARMS2, that interacts with properdin, three ARMS2 peptides encompassing all amino acids of ARMS2 were coated to a microtiter plate and incubated with properdin. Peptide 3 of ARMS2, but not peptides 1 or 2, showed binding to properdin (Fig. 5e). These results confirm interaction of ARMS2 with properdin and localize the interaction domain in the C-terminal part of ARMS2.

**Fig. 6:**
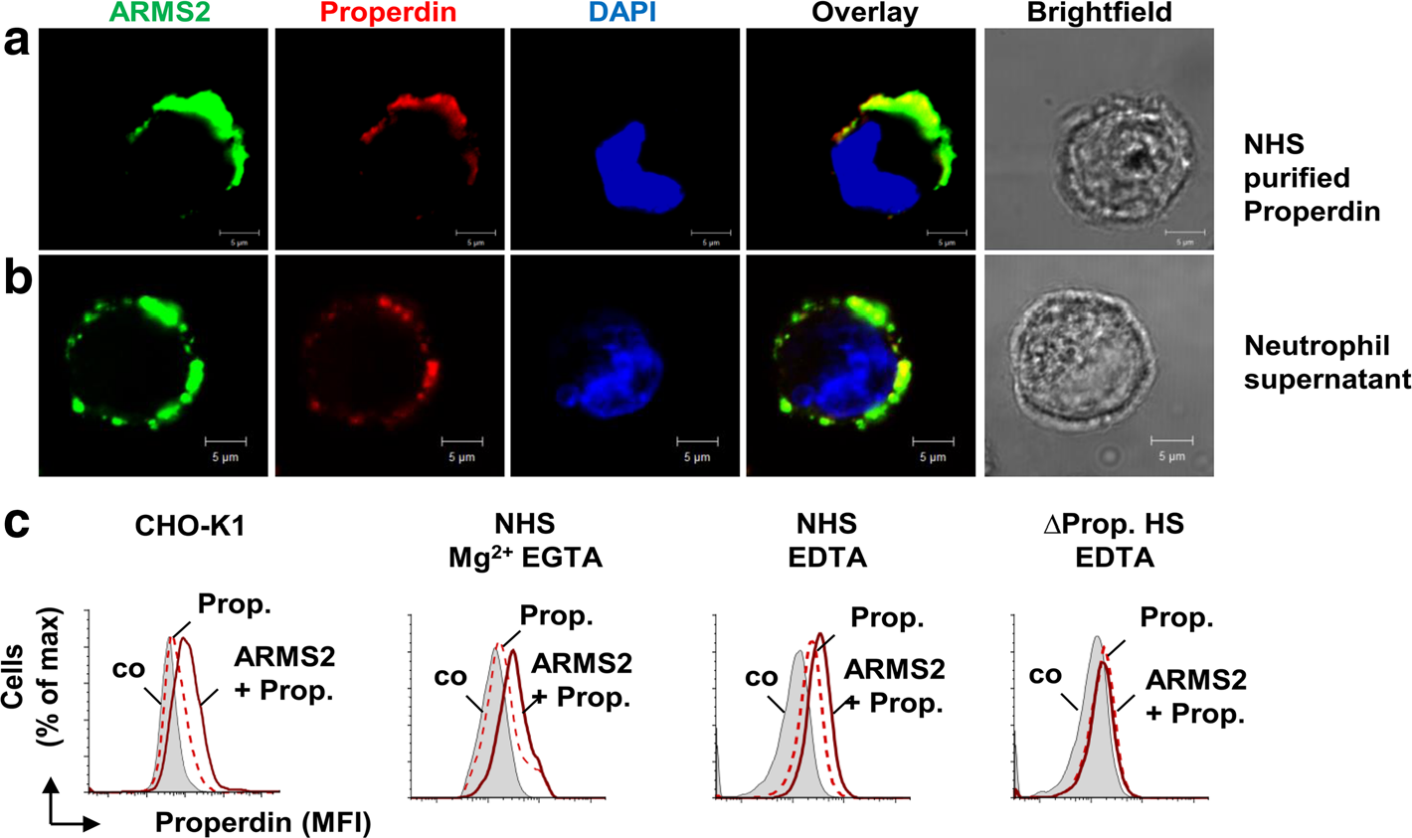
ARMS2 anchors the human complement activator properdin on cell surfaces. **a** Co-localization (*yellow*) of ARMS2 (*green*) and purified properdin from NHS (*red*) or (**b**) properdin from supernatant of human neutrophils (*red*) on CHO-K1 cells by laser scanning microscopy. Nuclei are stained with DAPI (*blue*). *Scale bar* 5 μm. Representative data from two independent experiments are shown. **c** Recruitment of purified (*left profile*) and endogenous properdin to ARMS2 on CHO-K1 cells. ARMS2 was attached to CHO-K1 cells and incubated for 1 h with purified properdin or in alternative pathway active (Mg^2+^ EGTA), complement inactive (EDTA), or properdin deficient HS as indicated. Properdin binding was determined by flow cytometry. Representative binding profiles of two independent experiments are shown. Background binding of the antibody to the cells is marked (*co*)

In NHS, properdin exists as trimers or tetramer complexes, and larger complexes are considered artifacts [30]. Therefore, binding of native serum-derived as well as neutrophil-derived properdin was evaluated to ARMS2 coated cells. CHO-K1 cells to which ARMS2 was attached were incubated in complement active NHS or in culture supernatant derived from human neutrophils. Following washing of the cells, ARMS2 and properdin were identified by confocal microscopy (Figs. 5b and 6a). ARMS2 and properdin, either derived from NHS or from supernatant of neutrophils, co-localized on the surface of CHO-K1 cells. Properdin binding to cell surfaces is often C3b dependent [32]. To demonstrate that ARMS2 recruits properdin directly and that ARMS2-properdin complexes form in the absence of C3b, binding of properdin, derived from complement active and inactive NHS, to ARMS2 coated CHO-K1 cells was followed by flow cytometry (Fig. 6c). ARMS2 coated to the cells bound purified properdin. ARMS2 also bound properdin from NHS treated with EGTA, which chelates Ca^2+^ and inhibits the classical complement pathway but leaves the alternative pathway intact. Also, when EDTA that inhibits Mg^2+^ and Ca^2+^ dependent C3 convertase formation was added to NHS to block C3b generation, properdin bound to surface attached ARMS2. Only when properdin depleted serum was used no properdin, binding to ARMS2 coated CHO-K1 cells was detected (Fig. 6d). Thus ARMS2-properdin complexes are also formed in the absence of C3b. Altogether, the data show that ARMS2 is a surface acting molecule that enhances C3b opsonization via binding of properdin. Thus, microglia cells with the non-risk *ARMS2* gene can enhance opsonization and phagocytosis of dead cells by the generation and secretion of ARMS2.

## Discussion

**Fig. 7:**
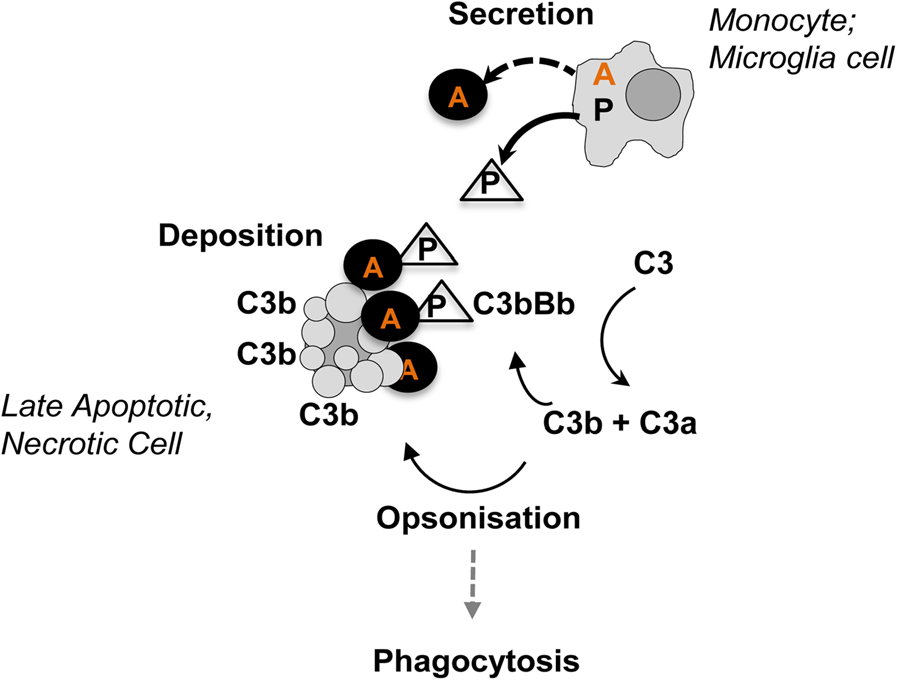
Schematic overview of ARMS2 functions. Human monocytes or microglia cells express ARMS2, which is likely secreted by a non-classical pathway and which attaches to late apoptotic and necrotic cells, recruits properdin and enhances C3b opsonization by complement activation. Opsonization of particles with C3b augments phagocytosis. AMD patients homozygous for the *ARMS2* risk haplotype (II/II) show ARMS2 protein deficiency in their monocytes

The present study demonstrates expression of ARMS2 in human monocytes as well as in human microglia cells. The ARMS2 expresssion was upregulated in monocytes upon oxidative stress. Deficiency of the ARMS2 protein was observed in monocytes homozygous of the AMD associated genetic polymorphism rs10490924 in the *ARMS2* gene. In addition, recombinant ARMS2 binds to surfaces of dead cells and when attached to cellular surfaces, ARMS2 anchors the complement activator properdin. By recruiting and anchoring properdin to cellular surfaces, ARMS2 enhances local complement activation and C3b surface opsonization. C3b/iC3b “tagging” of cells likely facilitates phagocytosis and ultimately removal of cellular debris (Fig. 7). This role of ARMS2 underlines the pivotal role innate immunity plays in retinal homeostasis.

*ARMS2* mRNA is expressed in human monocytes and also in stem cell induced human microglia cells, but not in murine macrophages. Expression in human but not in murine cells is in agreement with the relatively late evolutionary appearance of the *ARMS2* gene in humans and higher primates [15]. Using a new antiserum, which was generated by immunizing rabbits with recombinant ARMS2 proteins, endogenous ARMS2 protein expression was detected in monocytes derived from individuals with the wild type *ARMS2* genotype and also in the human monocytic cell line THP-1, which is heterozygous for the risk ARMS2 haplotype. Similarly, ARMS2 was immunoprecipitated from THP-1 cells with a novel monoclonal ARMS2 antibody. However, the ARMS2 concentration was very low indicating that a trigger is necessary for ARMS2 expression in the cells. Indeed, after blood monocytes were stressed with H_2_O_2_, ARMS2 expression was induced and detected also by Western blotting. Thus, ARMS2 is expressed upon oxidative stress in monocytes and likely also microglia cells, which is in agreement with oxidation specific epitopes on damaged cells in the retina which induce complement activation (3). The recombinant ARMS2 protein, when separated under reducing conditions, appeared as a band with a molecular mass of about 17 kDa, which was identified as ARMS2 by mass spectrometry. The whole deglycosylated, untagged recombinant protein showed a mass of 11.349 kDa, which is smaller than the calculated mass of 11.4 kDa. The difference is explained by pre-treatment of the protein (deglycosylation and removal of the histidine tag). However, deglycosylation of recombinant ARMS2 did not alter binding of ARMS2 to cells or interaction with proteins (data not shown).

*ARMS2* gene variations represented by two different polymorphisms, affect ARMS2 protein expression in monocytes. The risk variant represented by polymorphisms rs10490924 is linked with the indel mutation, which affects mRNA stability [12]. The second polymorphism, i.e., rs2736911, which is very rare in homozygous setting in Caucasians but more frequent in individuals of Chinese origin [33], generates a premature stop codon at position 38. Apparently, both polymorphisms rs10490924 and rs2736911 result in ARMS2 protein deficiency as shown in monocytes and retinal section stainings. These results confirm the recent reports demonstrating that the indel mutation in the 3′ untranslated region of the *ARMS2* gene leads to RNA instability [12, 25] and ARMS2 protein deficiency in placenta tissue, derived from a person with the ARMS2 risk genotype [12]. These data may help to resolve the contradictory views that exist over ARMS2 as a key effector protein in AMD and on the functional consequences of the polymorphism rs10490924 in *ARMS2* [34]. The polymorphism rs10490924 is in strong linkage disequilibrium with SNP rs1100638 in the *HTRA1* (high temperature requirement factor A1) gene [12, 34, 35] which makes effects by these polymorphisms indistinguishable in statistical analyses.

The innate immune system constantly monitors a spectrum of surfaces, including self, altered self and non-self. Potential targets are recognized on the basis of their characteristic surface molecular patterns. We here demonstrate that the recombinant ARMS2 protein like the complement regulator factor H binds preferentially to apoptotic and necrotic surfaces and identifies heparan sulfate as an ARMS2 ligand. Interestingly, surface-bound recombinant ARMS2, but not in fluid phase, serves as an anchor for the human complement activator properdin. ARMS2-properdin complexes formed at cellular surfaces enhanced C3 convertase formation and C3 convertases mediated C3b opsonization. Such marking of surfaces with C3 cleavage products accelerates recognition, uptake and clearance of debris by human phagocytes [21, 22], and binding of properdin to apoptotic cells enhances this process [28]. Notably, in addition to surface complement activation, complement regulators like factor H and C4bp are recruited to the surfaces to restrict the complement cascade and to inhibit inflammation and autoimmune reactions [3, 21, 22].

## Conclusions

The data presented suggest that ARMS2 assists in the clearance of cellular debris in the human retina. Recombinant ARMS2 distinguishes certain modified cells via surface glycosaminoglycan structures and initiates complement. Therefore, in case of ARMS2 deficiency complement-mediated opsonization, uptake and phagocytosis of dying cells are expected to be impaired. Over a long time, i.e., over years, such a reduced clearance may support accumulation of cellular debris in the retinal space and along Bruch’s membrane enhancing formation of drusen, the hallmark of AMD.

## Additional file

Additional file 1: Figure S1. Expression vector with ARMS2 coding sequence. (PDF 268 kb)

## Abbreviations

AMD: Age-related macular degeneration
ARMS2: Age-related maculopathy susceptibility 2
FACS: Fluorescence-activated cell sorting
iPSdM: Induced pluripotent stem cell-derived microglia
LSM: Laser scanning microscopy
PCR: Polymerase chain reaction
