# Supplementary material for: Age-related macular degeneration associated polymorphism rs10490924 in ARMS2 results in deficiency of a complement activator
Source: J Neuroinflammation. 2017 Jan 5;14:4. doi: 10.1186/s12974-016-0776-3 (PMC5234120; doi:10.1186/s12974-016-0776-3)
Supplement: Additional file 1: Figure S1. — Expression vector with ARMS2 coding sequence. (PDF 268 kb) [file 12974_2016_776_MOESM1_ESM.pdf]

```
ARMS2

ATG CTG CGC CTA TAC CCA GGA CCG ATG GTA ACT GAG GCG GAG GGG AAA GGA GGG CCT
 M   L   R   L   Y   P   G   P   M   V   T   E   A   E   G   K   G   G   P
GAG ATG GCA AGT CTG TCC TCC TCG GTG GTT CCT GTG TCC TTC ATT TCC ACT CTG CGA
 E   M   A   S   L   S   S   S   V   V   P   V   S   F   I   S   T   L   R
GAG TCT GTG CTG GAC CCT GGA GTT GGT GGA GAA GGA GCC AGT GAC AAG CAG AGG AGC
 E   S   V   L   D   P   G   V   G   G   E   G   A   S   D   K   Q   R   S
AAA CTG TCT TTA TCA CAC TCC ATG ATC CCA GCT GCT AAA ATC CAC ACT GAG CTC TGC
 K   L   S   L   S   H   S   M   I   P   A   A   K   I   H   T   E   L   C
TTA CCA GCC TTC TTC TCT CCT GCT GGA ACC CAG AGG AGG TTC CAG CAG CCT CAG CAC
 L   P   A   F   F   S   P   A   G   T   Q   R   R   F   Q   Q   P   Q   H
CAC CTG ACA CTG TCT ATC ATC CAC ACT GCA GCA AGG..(TGA)..GAC GAC GAC GAC AAA
 H   L   T   L   S   I   I   H   T   A   A   R..........D   D   D   D   K
                                                          enterokinase
CTT CTA GAA CAA AAA CTC ATC TCA GAA GAG GAT CTG AAT AGC GCC GTC GAG CAT CAT
 L   L   E   Q   K   L   I   S   E   E   D   L   N   S   A   V   D   H   H
                    C-myc epitope
CAT CAT CAT CAT TGA...
 H   H   H   H
histidine tag
```

Supplementary Figure 1: Expression vector with ARMS2 coding sequence

Recombinant ARMS2 is expressed via pPICZB expression vector (Thermo Fisher Scientific) in *Pichia pastoris* (X33 (Life Technologies). The ARMS2 protein is fused to a c-myc epitope (pink) and histidine tag (orange) for purification. The tag can be cleaved off by enterokinase (blue). The calculated mass of ARMS2 is 11.4 kDa and as fusion protein with tag 14.62 kDa.
